# Viscose-Derived Activated Carbons Fibers as Highly Efficient Adsorbents for Dimethoate Removal from Water

**DOI:** 10.3390/molecules27051477

**Published:** 2022-02-22

**Authors:** Ana Jocić, Stefan Breitenbach, Danica Bajuk-Bogdanović, Igor A. Pašti, Christoph Unterweger, Christian Fürst, Tamara Lazarević-Pašti

**Affiliations:** 1 VINČA Institute of Nuclear Sciences-National Institute of the Republic of Serbia, University of Belgrade, Mike Petrovica Alasa 12-14, 11000 Belgrade, Serbia; ana.jocic@vin.bg.ac.rs; 2 Wood K plus–Kompetenzzentrum Holz GmbH, Altenberger Strasse 69, 4040 Linz, Austria; s.breitenbach@wood-kplus.at (S.B.); c.unterweger@wood-kplus.at (C.U.); c.fuerst@wood-kplus.at (C.F.); 3 Institute of Chemical Technology of Inorganic Materials (TIM), Johannes Kepler University Linz, Altenberger Strasse 69, 4040 Linz, Austria; 4 University of Belgrade–Faculty of Physical Chemistry, Studentski trg 12-16, 11158 Belgrade, Serbia; danabb@ffh.bg.ac.rs (D.B.-B.); igor@ffh.bg.ac.rs (I.A.P.)

**Keywords:** water remediation, dimethoate removal, activated carbon fibers

## Abstract

Extensive use of pesticides resulting in their accumulation in the environment presents a hazard for their non-target species, including humans. Hence, efficient remediation strategies are needed, and, in this sense, adsorption is seen as the most straightforward approach. We have studied activated carbon fibers (ACFs) derived from viscose fibers impregnated with diammonium hydrogen phosphate (DAHP). By changing the amount of DAHP in the impregnation step, the chemical composition and textural properties of ACFs are effectively tuned, affecting their performance for dimethoate removal from water. The prepared ACFs effectively reduced the toxicity of treated water samples, both deionized water solutions and spiked tap water samples, under batch conditions and in dynamic filtration experiments. Using the results of physicochemical characterization and dimethoate adsorption measurements, multiple linear regression models were made to reliably predict performance towards dimethoate removal from water. These models can be used to quickly screen among larger sets of possible adsorbents and guide the development of novel, highly efficient adsorbents for dimethoate removal from water.

## 1. Introduction

Pesticides are widely used to improve agricultural production and control various pests and disease vectors in public health. However, despite their many benefits, excessive or improper application of pesticides in agricultural activity leads to the pollution of soils and aqueous environments, which may produce a range of hazardous effects to non-target species such as humans and animals [1].

Organophosphorus pesticides (OPs) are among the most commonly used compounds to control pests for both agricultural and residential applications. Their usage is still growing because of their high efficacy, broad spectrum of activity, multi-pest control capability, lack of pest resistance, and low cost [2]. However, the increased application of OPs leads to the pollution of land and water ecosystems and is a serious threat to human health due to the toxic nature of these compounds [2]. Their primary toxicity is associated with the irreversible inhibition of acetylcholinesterase enzyme (AChE) in the nervous system and blood, resulting in acetylcholine accumulation and, consequently, disrupted neurotransmission [3].

Dimethoate (*O*, *O*-Dimethyl*S*-[2-(methylamino)-2-oxoethyl] phosphorodithioate) is a very effective OP insecticide and acaricide both in agriculture (the control of crop pests in soil and on foliage) and non-agricultural applications (the control of mosquitoes, flies, cockroaches, termite) [4]. WHO classification places dimethoate as a second-class pesticide with moderate toxicity [5]. However, their metabolites are secondary pollution problems under optimum environmental conditions and the influence of microbes, chemical or physical agencies. Dimethoate converts to many more toxic products than the primary pesticide [3]. Specifically, dimethoate can be transformed into its oxo-form, omethoate, during the drinking water disinfection processes, which is more toxic than dimethoate [6]. Due to potentially dangerous effects on human health, WHO has set a guideline value of 6 μgdm^−3^ for dimethoate in water [7].

Due to the high toxicity and tremendous effect of OPs on the ecosystem, developing effective and economically feasible methods for removing the environment is a global issue. Therefore, different methods have been reported for OPs remediation, such as bioremediation [4,8], photodegradation [9,10,11], membrane filtration [12] and adsorption techniques [13,14,15,16]. However, adsorption has been considered one of the most suitable techniques for OP removal from an aqueous medium due to its simplicity, cost-effectiveness, environment friendliness, and the possibility of scaling up the process; it is widely used not only for OPs but also for other contaminants [16,17].

Activated carbon fibers (ACFs) are suitable for contaminant adsorption because of their unique and well-developed structural properties. They have a high specific surface area (reaching up to 3000 m^2^ g^−1^) and uniform microporosity [18]. Moreover, they can possess various surface functional groups with an affinity for different adsorbates, so these features provide them with high adsorption kinetics and capacities [19]. Previous studies have proved the ability of ACF to remove numerous pollutants contained in water, namely pesticides [20], metal ions [21], and organic micropollutants [22]. Moreover, the adsorption capacity of pesticides by ACF were found to be significantly higher than that of granular activated carbon due to a smaller diameter of fibers, which leads to a larger surface area accessible to pesticides, as well as narrower micropore size distribution, which enables a lower mass transfer resistance [23,24]. The structural and chemical characteristics and, consequently, adsorption properties of the ACF mainly depend on the used precursor and the applied production methods. Therefore, it is of great importance to find suitable raw materials that contain favorable characteristics and that, at the same time, are economically and ecologically attractive. Viscose fibers are appropriate precursors for ACF preparation due to their fibrous structure, good processability, bio-based origin, and availability with good and constant quality. Moreover, using impregnation agents during the preparation of viscose-based ACFs can significantly enhance the structural characteristics of the produced material and reduce production costs by increasing yields [18].

In this contribution, ACFs prepared from viscose fibers impregnated using diammonium hydrogen phosphate (DAHP) [18] were investigated as adsorbents for dimethoate removal in detail. Previously, we had shown that these ACFs were capable for OPs removal, particularly focusing on chlorpyrifos removal [25]. Using DAHP in a wide concentration range, chemical composition and textural properties of produced ACFs were effectively tuned, while fibrous morphology was preserved. Produced ACFs were used as adsorbents for dimethoate in water and displayed high efficiency for its removal under both batch and dynamic conditions. Materials performance and guidelines for developing novel materials for dimethoate removal are discussed in terms of their physicochemical properties.

## 2. Results and Discussion

### 2.1. Materials Morphology and Chemical Composition Using EDX

**Figure 1:**
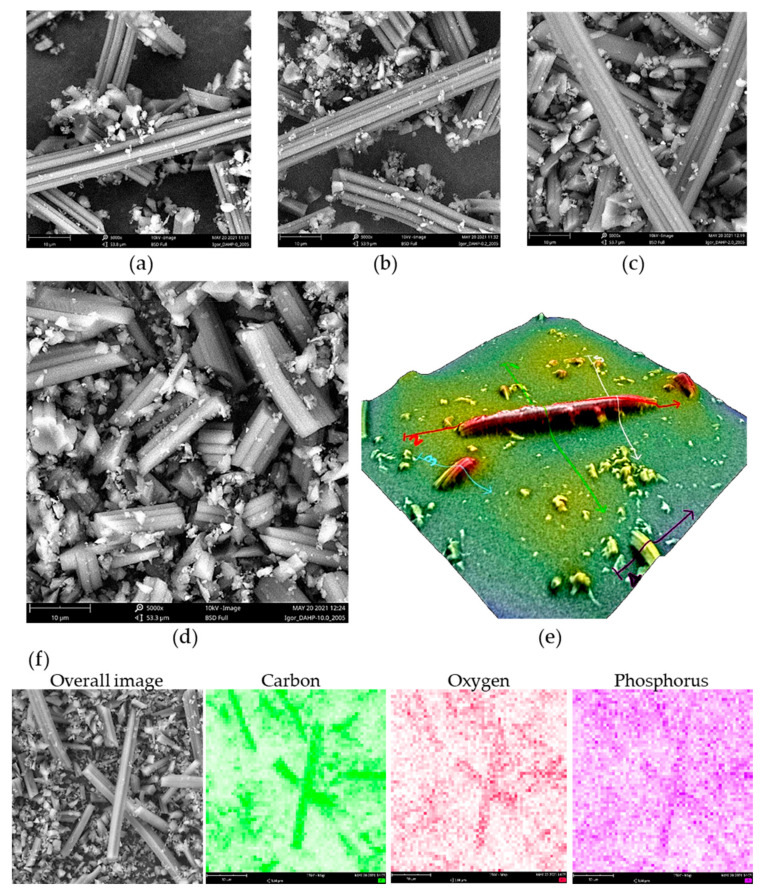
SEM micrographs of (**a**) DAHP-0, (**b**) DAHP-0.2, (**c**) DAHP-5.0, (**d**) DAHP-10 (magnification × 5000, field of view 53.6 μm, scale bar 10 μm), and (**e**) 3D reconstruction of individual small fiber debris showing diameter of ACFs of roughly 8 μm (magnification × 5000), (**f**) EDX mapping for sample DAHP-2.5 (magnification × 5000, field of view 53.6 μm, scale bar 10 μm).

Using SEM, we have found that the morphology of all prepared ACF samples is the same and reflects, besides a shrinkage of approx. 30%, the morphology of precursor viscose fibers, in agreement with our previous findings [26]. SEM micrographs are presented in Figure 1. Certain ACFs broke into smaller pieces during the milling step, while some intact ACFs were also seen, with lengths reaching 200 μm and approx. 8 μm in diameter (Figure 1e). Small debris seen in SEM images resulted from the milling process and have the same elemental content as larger particles. The formation of small debris is likely due to natural inhomogeneities in precursor viscose fibers, resulting in local differences in mechanical properties upon carbonization and activation.

In contrast to morphology, the chemical composition of ACFs was affected by the loading of DAHP during the impregnation. Using EDX, we observed an increasing trend in the P content in ACFs, as summarized in Table 1. The results followed the trend reported previously for ACFs produced from DAHP-impregnated viscose fibers, but O content was significantly greater [18]. This might be due to the adhesive tape used to paste the powders onto the SEM holder, which contained a large fraction of O (and additional C), but this effect produced a constant bias in oxygen content determined in the samples. Thus, the results can be considered indicative of elemental composition trends and not taken as the absolute values.

It is important to note that the distributions of C, O, and P were rather uniform in the samples, as seen from the EDX mapping under low magnification and mapping on individual fibers (Figure 1 and Figure S1, Supplementary File S1). We also found that P incorporation was observed not only on the surface but also in the inner part of ACFs, as EDX confirmed the presence of P along the cross-section of ACFs formed during the milling of ACFs (Figure S2, Supplementary File S1). This indicates that DAHP entered the pores of precursor viscose fibers during the impregnation step and resulted in the incorporation of P throughout the entire volume of ACFs. It is important to note that the pore system of non-carbonized viscose fibers is generally poorly developed, reaching only a few m^2^ g^−1^ and dominant micropores [27], but, apparently, enables the penetration of DAHP from the solution phase to the interior of fibers.

### 2.2. Textural Properties

**Figure 2:**
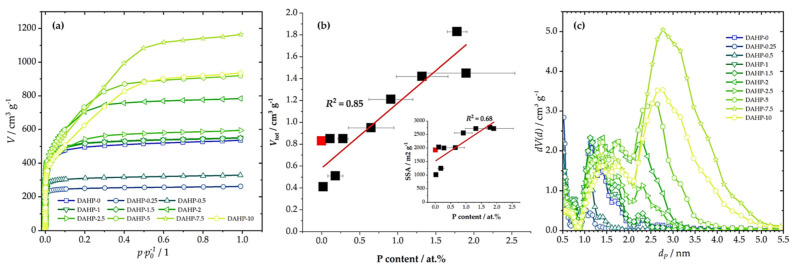
N_2_ adsorption isotherms (**a**), the correlation between *V*_tot_ and P content (inset gives the correlation between SSA and P content; lines give linear fit); red square stands for DAHP-0 sample (**b**), and derived PSDs (**c**) for the studied samples.

The specific surface area (SSA) and total pore volume (*V*_tot_) for each sample determined using N_2_ adsorption measurements (Figure 2a) are shown in Table 1. The first P-containing sample in the series showed a large decrease in SSA compared to the ACFs produced without DAHP impregnation. However, upon increasing the DAHP concentration, SSA and *V*_tot_ increased. One can consider that the sample DAHP-2 was an outlier, but, according to the EDX results, this sample also contained a larger fraction of P incorporated in the structure. Thus, there was a rather good correlation between *V*_tot_ and SSA and P content (Figure 2b). While our focus was not on synthesizing ACFs, a pronounced shift of pore sizes from micro to mesopores (Figure 2c) with increasing DAHP concentration should be emphasized. This finding cannot be ascribed solely to the effects of DAHP, as we have previously shown that after carbonization of impregnated viscose fibers (4% DAHP), SSA reached 292 m^2^ g^−1^, and the material was dominantly microporous [26]. On the other hand, the mesopore system developed after activation with CO_2_ and SSA increased to 2245 m^2^ g^−1^. Hence, this had to be a combined effect of DAHP and CO_2_ activation, as analogous development of the mesopore system was not present in the case of ACFs derived from ammonium sulfate-impregnated viscose fibers. In this case, CO_2_ activation only developed a micropore system [26], as in non-impregnated carbonized viscose fibers (Figure 2).

This correlation is very important considering the impact of different properties of studied materials on the adsorption performance, as will be discussed further. In addition, we also note that SSA and *V*_tot_ are in excellent correlation, although, at the high SSA side, this correlation deviated from a straight line. This is because the increase of the P content caused the shift of the dominant pore range from microporous to mesoporous (Figure 2c). Hence, increasing P content (and O content simultaneously) resulted in a gradual increase in pore sizes.

Here we do not discuss in detail the mechanism of DAHP effects on the properties of the final ACFs, but refer to the original work regarding the synthesis of these fibers [18]. In the context of the present work, the important point is that DAHP significantly increased the yield of produced ACFs, in addition to the tuning of the elemental content and the pore size distribution, as will be discussed further.

### 2.3. Raman and FTIR Spectroscopy

**Figure 3:**
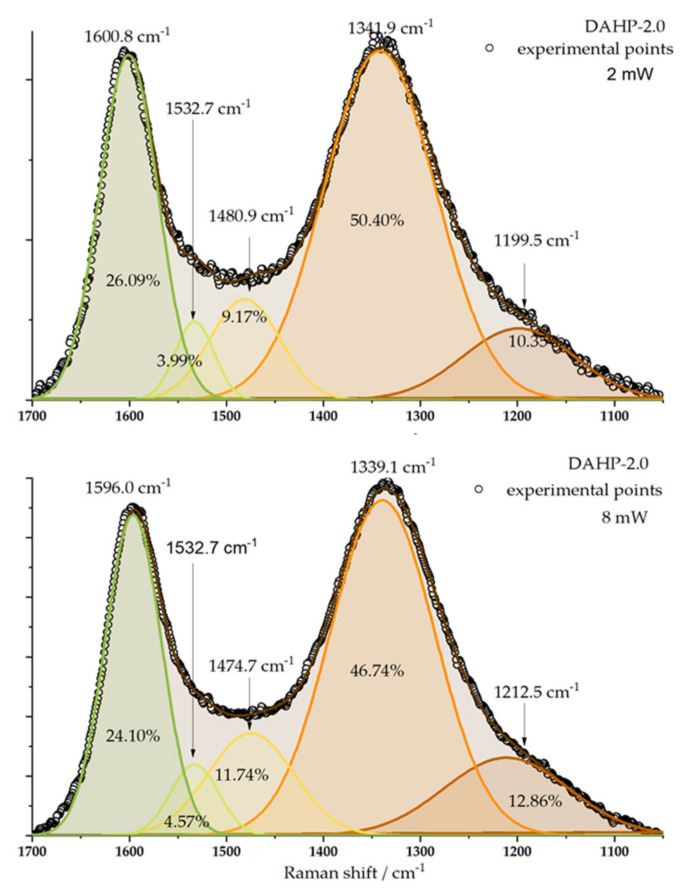
Deconvolution of Raman spectra of DAHP-2.0 sample is presented for the laser power of 2 and 8 mW. Positions of individual bands and their relative contributions to the overall signal in the considered wavenumber range are indicated.

Raman spectra were recorded for the studied samples (Figure S3, Supplementary File S1). Interestingly, despite the different chemical compositions of the samples, we could not differentiate between the recorded spectra in such a way as to outline any particular trend. However, characteristic bands around 1340 and 1580 cm^−1^ were clearly seen (Figure 3). These were assigned to D (originating from sp^3^-hybridized carbon) and G band (originating from the sp^2^-hybridized graphitic phase of the carbon) [28]. The laser power change from 2 to 8 mW only slightly affected the D and G band intensities ratio but did not manage to differentiate between the studied samples to an appreciable level. Taking the sample DAHP-2.0 as an example, the Raman signal of D and G band regions can be split into five components [28,29], and, if the peak areas are used to evaluate the *I*_D_/*I*_G_ ratio, the result is 1.93 for 2 mW and 1.94 mW for 8 mW laser power. However, the numbers themselves indicate a significant number of defects in the ACFs structure.

FTIR spectra (Figure S4, Supplementary File S1) showed a clear evolution with increasing P content. The assignation was carried out according to [30,31], suggesting that, with the increase of the P content, bands associated with C=C vibrations (1580 cm^−1^) and particularly vibrations of O containing groups (–C–O–C–, –COOH, –O–, C–O, and others), in the range between 1250 and 1000 cm^−1^ become more pronounced. Moreover, the bands of the mentioned O-containing groups also fall in the range of wavenumbers at which the vibrations of the C–P moieties can be found (around 1200 cm^−1^, while the C–P can also appear around 900 cm^−1^) in organic P-containing molecules [32].

### 2.4. Dimethoate Removal from Aqueous Solutions

**Figure 4:**
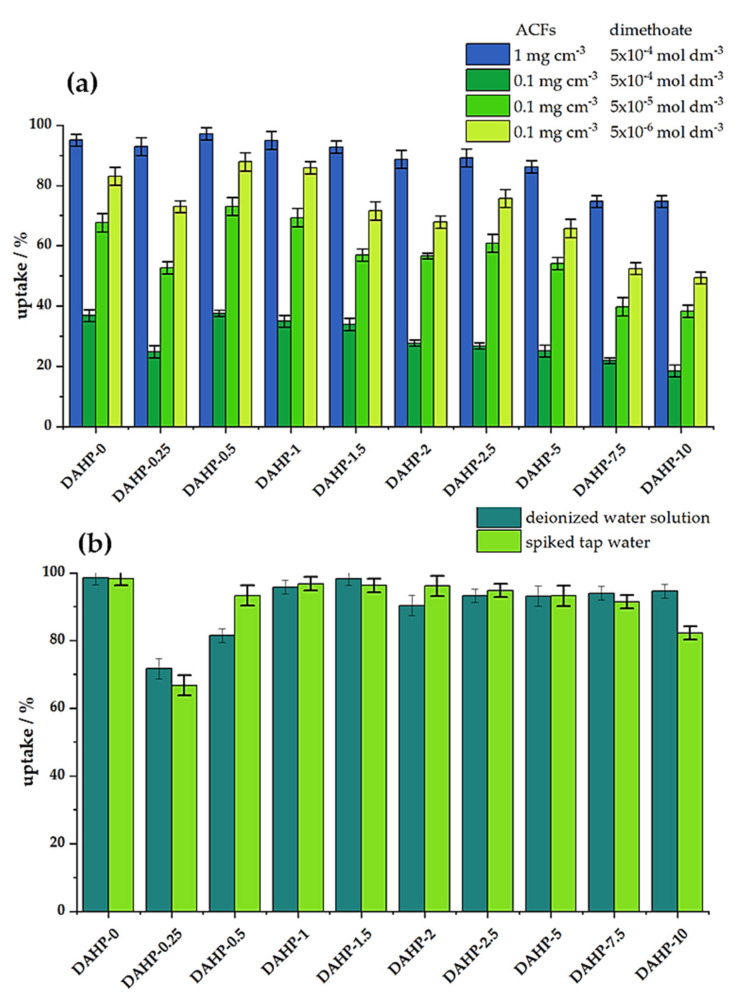
(**a**) Dimethoate removal (in %) under batch conditions for different concentrations of ACFs and dimethoate (20 min equilibration time, 25 °C); (**b**) Dimethoate removal under dynamic conditions. Filters were filled with 1mg of ACFs, and then 1 cm^−3^ of 5 × 10^−4^ mol dm^−3^ dimethoate solution was filtered for 1 min through them (25 °C).

Following the physicochemical characterization of the ACFs, we studied dimethoate removal under bath (equilibrium) conditions and under dynamic conditions (filtration). Preliminary experiments showed that 20 min of equilibration time was sufficient to reach steady conditions. The results for the adsorbent concentration of 1 mg cm^−3^ and dimethoate concentration of 5 × 10^−4^ mol dm^−3^ are shown in Figure 4a. We also tested dimethoate removal for a lower concentration of ACFs (0.1 mg cm^−3^) and different concentrations of dimethoate (5 × 10^−4^, 5 × 10^−5^, and 5 × 10^−6^ moldm^−3^), Figure 4a. We observed that the trends were preserved in all the cases: the best performance was seen for materials with low P content, reaching a maximum for the DAHP-0.5 sample, taking 97% of dimethoate from the 5 × 10^−4^ mol dm^−3^ solution (ACFs concentration 1.0 mg cm^−3^). Upon increasing the P content, dimethoate uptake decreased and fell below 80% for the DAHP-7.5 and DAHP-10 samples. As these samples have the highest SSAs of all the studied ACFs; this is a very clear indication that SSA does not solely determine the performance of dimethoate removal from water under equilibrium conditions. Nevertheless, these materials are not bad dimethoate adsorbents but perform much lower than the other ACFs in the studied series. When the amount of adsorbed dimethoate is expressed as the adsorption capacity, giving the mass of dimethoate adsorbed per unit mass of ACFs, these values range between 111 and 86 mg g^−1^ (1 mg cm^−3^ of ACFs and dimethoate concentration of 5 × 10^−4^ mol dm^−3^). For the experiments performed with 0.1 mg cm^−3^ of ACFs, dimethoate adsorption isotherms pointed to adsorption capacities above 400 mg g^−1^ for the best performing materials in the series (Figure S5, Supplementary File S1). It is important to note that the best performance of ACFs impregnated with lower concentrations of DAHP was extremely important from a practical point. Namely, the total yield of ACFs after carbonization and activation steps was maximized for low concentrations of DAHP as an impregnation agent (reaching 20%, [18]). When no DAHP was used, the total yield was only 1.1%, with critical losses during the activation step; when high concentrations of DAHP were used, yields ranged from 6 to 10%.

Under dynamic conditions, materials performed similarly in deionized and spiked tap water samples, suggesting that matrix effects are rather small (Figure 4b). In this case, the lowest performance was observed for the sample DAHP-0.25, which had the lowest SSA (Table 1). It was followed by DAHP-0.5 and DAHP-10 samples, while other samples had dimethoate uptake above 90% in both deionized water and spiked tap water solutions. However, while the complexity of dimethoate removal under dynamic conditions was much higher compared to the equilibrium adsorption conditions, the obtained results suggest, again, that SSA was not the dominant factor for dimethoate removal.

It was necessary to verify the reduced toxicity of treated water samples and to exclude possible conversion of dimethoate to more toxic oxo-form. This was also important in the context of the recently demonstrated reaction between dimethoate and O-rich carbon using graphene oxide as an example [33]. The experiments were carried out using spiked tap water samples, and an efficient reduction of water toxicity was clearly seen (Table 2). As higher AChE inhibition level correlated with lower dimethoate uptake, the toxicity measurements were in excellent correlation with the bath and filtration measurements. Under dynamic conditions, the most effective ACFs were DAHP-0, while, under batch conditions, DAHP-0.5 led the series. Both samples completely removed any water toxicity upon the treatment under mentioned conditions.

### 2.5. Adsorption Isotherms for Dimethoate Removal

To better understand the adsorption of dimethoate onto studied ACFs, we fitted our experimental data obtained under equilibrium conditions into several frequently used adsorption isotherms (Freundlich, Langmuir, and Dubinin–Radushkevich). The linearized forms of these isotherms are [34]:

(1) $$\log q_{e}=\log K_{f}+\frac{1}{n}\log C_{e}\;(\mathrm{Freundlich})$$

(2) $$\frac{C_{e}}{q_{e}}=\frac{1}{bq_{\max}}+\frac{C_{e}}{q_{\max}}\;\;(\mathrm{Langmuir})$$

(3) $$\ln q_{e}=\ln q_{\mathrm{DR}}-K_{\mathrm{DR}}{Ɛ}^{2}\;(\mathrm{Dubinin}–\mathrm{Radushkevich})$$

The used parameters are: *q_e_* (mgg^−1^) equilibrium adsorption capacity, *C_e_* (mg dm^−3^) equilibrium adsorbate concentration, *K_f_* (mg g^−1^ (mg dm^−3^)^1/*n*^) and Freundlich constants, *q*_max_ (mg g^−1^) theoretical maximum adsorption capacity of the monolayer, *b* (dm^3^mg^−1^) Langmuir constant, *q*_DR_ maximum adsorption capacity, *K*_DR_ (mol^2^ J^−2^) constant associated with the mean free adsorption energy per mole of adsorbent, *E* free adsorption energy per mole adsorbent *E* = (−2*K*_DR_)^−1/2^, *ε* = RTln (1 + 1/*C_e_*). We used linearized forms of isotherms to fit the experimental data and user *R*^2^ to measure the quality of the fit. The obtained results are summarized in Table 3.

According to Langmuir’s model, dimethoate molecules should be adsorbed on an energetically homogeneous surface, in a monolayer, without interactions between the adsorbed molecules. All active centers were energy equivalent, and equilibrium was achieved by forming a monolayer of adsorbents on the adsorbate surface. Constant *b* had the highest value in the adsorption of dimethoate on DAHP-0.5 (0.010 dm^3^ mg^−1^). Higher values of this constant indicated an increased affinity of given ACFs towards dimethoate. However, the Langmuir model gave extremely high values of the maximum adsorption capacity of the monolayer (*q*_max_ up to 4000 mg g^−1^), which was not in accordance with the literature data for pesticide adsorption on carbon materials. Therefore, despite high *R*^2^ values, this model was not suitable for describing experimental data.

Freundlich’s model predicted adsorption on an energetically heterogeneous surface, where the adsorbed molecules interacted with each other. If *n* = 1, the adsorption followed the linear function. If *n* < 1, the adsorption was unfavorable, and if *n* > 1, the adsorption was favored. The values of *n* obtained by fit were all above 1, so the affinity of dimethoate for the adsorbent was high and the highest for DAHP-0.5.

The Dubinin–Radushkevich (DR) model could explain the nature of the adsorption process, that is, whether physisorption or chemisorption predominated on the adsorbent surfaces. Namely, the free adsorption energy *E* could be calculated from the DR equation, and, if higher than 8 kJ mol^−1^, chemisorption prevailed in the system, while lower values indicated physisorption. In the tested systems, the obtained *E* values were significantly lower than 8 kJ mol^−1^. Therefore, it could be concluded that physisorption was dominant in all the studied cases. The values of *q*_DR_ ranged from 99.2 to 196.4 mg g^−1,^ depending on the observed material. These values were of the order of others reported in the literature so far [13]. Nevertheless, it should be noted that the DR model had lower values of correlation coefficients (0.799 < *R*^2^ < 0.84), so the exact values of adsorption parameters should be taken with care. However, the conclusion regarding dominant physisorption was valid, also considering a good fit using Freundlich isotherm.

### 2.6. Materials Properties and Their Link to Dimethoate Removal

Elucidating materials’ properties–performance relations is always a challenge, but it is utterly important to improve materials for further given applications. However, parametric space used to describe different materials in terms of their properties and performance can be huge; it is essential to reduce it somehow. Even if this can be achieved only partially, making quantitative links between all these parameters can be rather useful for developing new materials. The approaches found in the literature differed by levels of complexity and sophistication, considering both mathematical models and the depth of materials characterization [35], but, here, we wanted to make it as simple as possible. First, we focused on finding the links between materials’ properties and performance and then on the physical justification of these relations.

**Figure 5:**
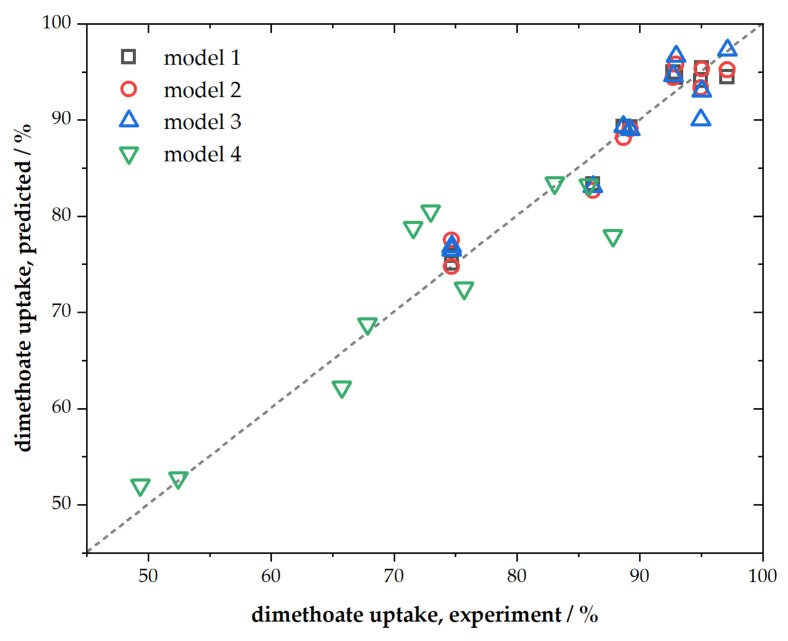
Parity plot for presented linear regression models.

Presented ACFs were characterized by their chemical composition, textural properties, spectroscopic characteristics, and performance towards dimethoate removal. We chose simple multiple linear regression to link dimethoate removal under different conditions (as dependent variable) with materials’ chemical composition (C, O, and P content) and textural properties (SSA and *V*_tot_) as independent variables. The regression analysis results are summarized in Table 4, while parity plots are given in Figure 5.

As can be seen, when all mentioned independent variables were taken into account, the fit was very good (*R*^2^ = 0.9994, model No. 1, Table 4) for the conditions corresponding to a higher adsorbent and dimethoate concentration. The fit was slightly poorer for the conditions corresponding to lower concentrations of dimethoate and ACFs but still of rather high quality (model No. 4, Table 4). However, when it came to rapid screening of materials as potential candidates for dimethoate removal, further reduction in the number of independent variables was desirable. As we have shown that SSA and *V*_tot_ mutually scaled, one of these variables could be considered redundant (better scaling between them means that this approximation will work better). Hence, when we removed SSA from the fit, we still predicted rather well the dimethoate uptake (model No. 2, Table 4). Further reduction was also possible considering that P content scaled with SSA and *V*_tot_, and also that the sum of C, O, and P content equaled unity. In this case, using only three independent variables (C and O content and *V*_tot_), we, again, obtained very good linear regression with *R*^2^ = 0.998 (model No. 3, Table 4). The possibility to exclude P content from the fit did not mean that it was irrelevant; its presence tuned the total pore volume.

Moreover, it is very interesting that SSA did not have one of the leading roles in determining dimethoate uptake (as also directly seen from the batch and filtration experiments), particularly when considering that high SSA ACFs also had larger pores (Figure 2b). However, with the increase of SSA (and *V*_tot_, Table 1), O content also increased (Table 1). This made the surface more hydrophilic and strongly solvated (hydrated). Considering that dimethoate was physisorbed on studied ACFs surfaces and that its solubility in water was relatively low, it was expected that weak dimethoate physisorption could not compensate (energetically) breaking of the solvation layer of ACFs. Hence, its uptake decreased as the surface became more hydrophilic. Taking the lateral size of dimethoate molecule (Figure S6, Supplementary File S1), it can be concluded that dimethoate can enter the pores below 0.65 nm, explaining adsorption on materials that were dominantly microporous and that had lower SSA than other samples (the ones obtained with low DAHP concentration). Assuming that the entire SSA can be covered by dimethoate molecules, for the DAHP-0.5 sample, one would expect an adsorption capacity of 892 mg g^−1^, far from that observed experimentally. In the case of the DAHP-10 sample, the same logic gives an adsorption capacity of 1940 mg g^−1^. This estimate shows that only a fraction of the surface is active for dimethoate removal; we suggest this was part of the carbon surface with lowered hydrophilicity so that extensive surface solvation did not block dimethoate adsorption. However, as dimethoate is an aliphatic polar molecule, it is suggested that these surface domains were not the ones with fully restored sp^2^ graphitic systems. It is more likely that surface functional groups such as –C–O–C–, –COOH, –O–, C–O, observed by FTIR, contributed to dimethoate adsorption via dispersion interactions (as expected from the results of Section 2.5), but at the domains where their concentration was not very high. These parts could be located in the ACFs pore system developed during the activation process as, under these conditions, extensive surface oxidation was not expected. In fact, we previously observed a slight decrease in oxygen content during the activation of DAHP-impregnated carbon fibers [26].

To further elaborate possible dimethoate-ACFs interactions, we used semi-empirical quantum chemical calculations. The surface model was constructed to contain a domain of an intact sp^2^ hybridized system and a part where oxygen functional groups (we opted for OH groups) were clustered, in line with refs. [36,37]. Water was added as solvent implicitly (see Section 3.5 for details).

**Figure 6:**
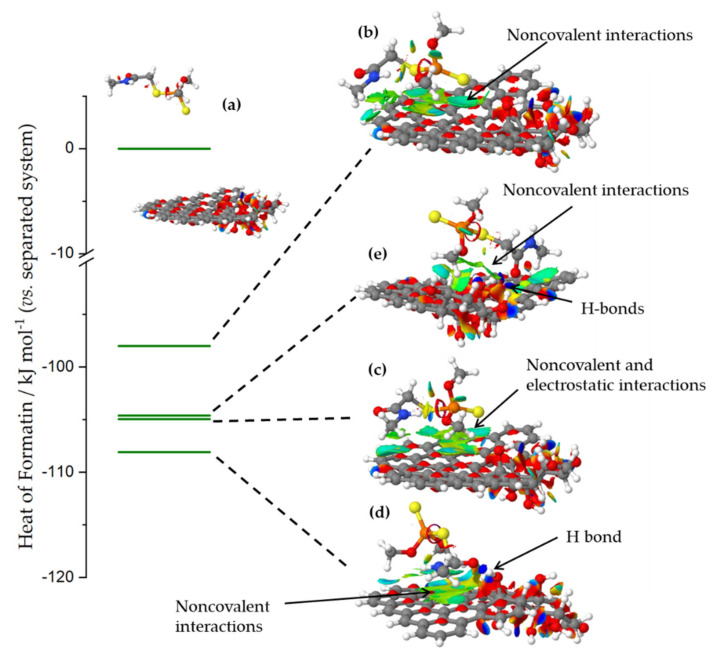
The heat of formation of different dimethoate–carbon surface configurations, relative to the system where dimethoate and carbon surface were separated so that no interactions occur. The systems were denoted (**a**–**e**) according to the order of discussion in the text (for the explanation check the text). Isosurfaces present the domains where non-covalent interactions were operative. The domains where different interactions were observed were indicated. The most stable configuration corresponded to the H-bond formed via carbonyl group in the dimethoate molecule, and additional stabilization took place through non-covalent interactions with the basal plane.

When dimethoate and carbon surface models were separated so that there was no interactions between them (Figure 6a), the system stabilization due to the presence of solvent was −2.81 eV. When dimethoate was brought into contact with the sp^2^-part of the carbon surface, the heat of formation of the systems was reduced compared to the separated system due to the non-covalent interactions with the basal plane, while stabilization due to the solvent was −2.22 eV (Figure 6b). However, further reduction of the heat of formation of the system was seen if dimethoate could interact with OH surface groups through the electrostatic or dispersion interactions or the formation (Figure 6c) or H-bonds via the carbonyl group of the dimethoate molecule (Figure 6d). In these cases, the interactions between dimethoate and surface functional groups stabilized the interaction, although system stabilization due to solvent was lower, roughly −2.05 eV in both cases. Non-covalent interactions between dimethoate and carbon surface provided additional stabilization of the system. In line with the previous discussion, it was important to note that the interaction was less favored if dimethoate was brought into contact with the OH groups cluster (Figure 6e). This was due to the desolvation of OH groups and disruption of the H-bond network, which stabilized the OH groups cluster, an energy penalty that cannot be compensated by new H-bonds formed between dimethoate molecule and the surface functional groups. Thus, we concluded that dimethoate was dominantly adsorbed at the surface where oxygen functional groups were present, but not at a high concentration, so that bonding could be achieved through electrostatic interactions and H-bond formation, but without a large impact on the solvation and inter-functional groups interactions.

While connecting material properties to dimethoate uptake can be carried out relatively easily using multiple linear regression, linking material properties with synthesis conditions could be much more difficult. Therefore, it would likely require more sophisticated approaches such as machine learning. However, if such relations were established, this would mean that one could set up a model for materials designed for dimethoate removal with desired performance.

### 2.7. Re-Use of DAHP Adsorbents

**Figure 7:**
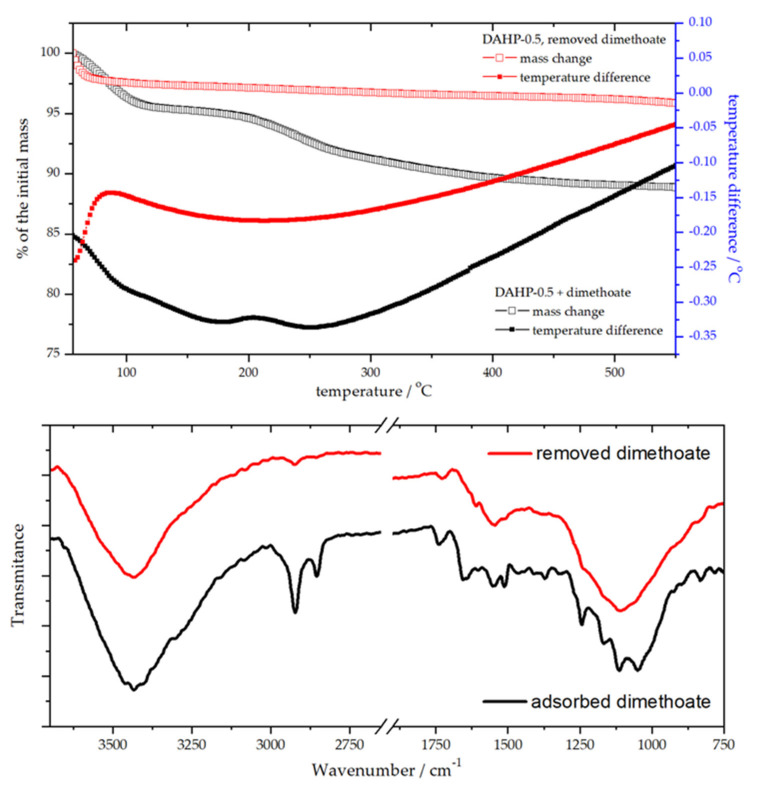
TG-DTA analysis of DAHP-0.5 before and after removing adsorbed dimethoate (**upper panel**) and the corresponding FTIR spectra (**lower panel**).

Natural questions regarding the use and re-use of DAHP adsorbents were whether phosphorous compound used for the impregnation could be released during the water treatment and whether adsorbent could be regenerated after the use. First, we confirmed no change in the phosphorus concentration in the adsorbent during the adsorption experiment using EDX. In fact, the ratios of elements present in DAHP-X samples had not changed during 6 months of being dispersed in water (see also Section 3.1). Next, for this purpose of adsorbent re-use, we investigated the regeneration of the best-performing material in the series (batch conditions), DAHP-0.5, using thermogravimetric (TG) and differential thermal analysis (DTA), combined with FTIR analysis before and after adsorbent regeneration (Figure 7).

The adsorbent regeneration was achieved by heating up to 550 °C, which effectively removed adsorbed dimethoate (Figure 7). TG-DTA curves showed endothermic processes of dimethoate removal. In contrast, for regenerated DAHP-0.5, only adsorbed water removal was seen at temperatures below 100 °C. Dimethoate removal from the adsorbent surface was also clearly seen from the FTIR spectra of DAHP-0.5 before and after regeneration of the adsorbent (Figure 7). However, the TG-DTA analysis revealed that dimethoate was removed in two main steps, one up to 100 °C and the second one up to 200 °C, with further removal of more strongly bound dimethoate at higher temperatures. This likely corresponds to dimethoate binding to weakly and strongly binding sites on the ACF surface, depending on the concentration of the oxygen functional groups, as discussed in the previous section, or, possibly, removal of dimethoate from inside the pores of DAHP-0.5. After regeneration, dimethoate uptake by regenerated adsorbent was found to be (96 ± 3) % (adsorbent 1 mg cm^−3^, dimethoate 5 × 10^−4^ mol dm^−3^).

## 3. Materials and Methods

### 3.1. Materials Synthesis

In the first step, viscose fibers (1.7 dtex, 38 mm) were dried for 24 h at 90 °C and then impregnated for 15 min in different solutions of DAHP in deionized water. The concentrations ranged from 0.0–75.7 mmol dm^−3^, matching 0.0–10.0% DAHP in distilled water. Upon impregnation, fibers were spin-dried for 15 min and then stored in a drying cabinet at 90 °C for 24 h. Next, carbonization was performed in a chamber furnace (HTK8, Gero, Germany) under a nitrogen atmosphere. The heating rate was 1.0 °C min^−1^, and upon reaching 850 °C, they were held isothermal for 20 min. Finally, the carbonized fibers were activated in a rotary kiln at 870 °C for 165 min in a CO_2_-flow of 80 dm^3^ h^−1^. Produced ACFs were used as synthesized without additional washing. Previous trials have shown that this additional process step is not needed due to the use of highly pure precursor fibers and an impregnation agent that did not introduce soluble components or change the pH value of the result ACF, in contrast to other chemical activation methods using, e.g., KOH or ZnCl_2_ [38,39]. The samples are noted as DAHP-X, where X stands for the concentration of DAHP used in the impregnation step.

### 3.2. Materials Characterization

The morphology of the ACF samples was investigated using a scanning electron microscope PhenomProX (Thermo Fisher Scientific, Waltham, MA, USA).

The specific surface area and textural structures of the obtained ACFs were analyzed by N_2_ isothermal adsorption (−196.15 °C) on a gas sorption system (AutosorbiQ, Quantachrome Instruments, Ashland, VA, USA). The samples were de-gassed for at least 2 h at 200 °C before the analysis. The specific surface area and derived pore size distribution (PSD) were calculated using the method of Brunauer–Emmett–Teller (BET) and the non-local density functional theory (NLDFT), respectively.

The Raman spectra of the samples were recorded on DXR Raman microscope (Thermo Fisher Scientific, Waltham, MA, USA). The samples were excited by the 532 nm emission line of a diode laser with 2 and 8 mW of power focused on a 2.1 μm spot on the surface of the sample. The spectrum was obtained as an average of three measurements on different spots on each sample (10 exposures, 10 s each, per place).

The FTIR spectra were recorded on a Nicolet iS20 FT-IR spectrophotometer (Thermo Fisher Scientific, Waltham, MA, USA) using the KBr pellet technique in a wavenumber range from 4000 to 500 cm^−1^ with 64 scans and 4 cm^−1^ resolution. All FTIR spectra were shown after automated baseline correction (polynomial order: 2, number of iterations: 20) performed by OMNIC software (Thermo Fisher Scientific, Waltham, MA, USA).

### 3.3. Pesticide Adsorption Measurements

Batch adsorption experiments were carried out as follows. First, prepared ACFs were dispersed in double distilled water, upon which the desired amount of dimethoate stock solution (Pestanal, Sigma Aldrich, Denmark) was added to provide the targeted concentration of adsorbent and dimethoate. Then, the vessel containing the adsorbent+dimethoate mixture was placed on a laboratory shaker (Orbital Shaker-Incubator ES-20, Grant-Bio, Cambridgeshire, UK) at 25 °C for desired times. Afterward, the mixture was centrifuged for 10 min at 14,500× *g*, and the supernatant was filtered through the nylon filter (pore size 220 nm KX Syringe Filter, Kinesis, Cole Parmer, St. Neots, UK). The concentration of dimethoate after adsorption (*C*_eq_) was determined using ultra performance liquid chromatography (UPLC). Control experiments were performed in identical ways but without ACFs and confirmed no dimethoate degradation during the batch experiments. In addition, the pH of adsorbent+dimethoate dispersions was monitored during the experiments (Table S1, Supplementary File S1); we did not observe differences between the samples. This also indicated that the differences in dimethoate removal efficiency were not due to chemical decomposition but due to the adsorption on studied samples.

To perform adsorption measurements under dynamic conditions, commercial nylon membrane filters (pore size 220 nm KX Syringe Filter, Kinesis, Cole Parmer, St. Neots, UK) were modified to include the adsorbent layer, as described in ref. [13]. The desired amount of each ACF sample was dispersed in 1.5 cm^3^ of deionized water and injected into the commercial filter. Then, the solvent was removed from the ACFs-modified filter using compressed air. Pesticide solution was run through the modified filter for 1 min. The filtrate was subjected to UPLC analysis to determine pesticide concentration after filtering. It was checked that dimethoate removal was not due to the nylon membrane by comparing pesticide concentrations before and after the filtration through the non-modified filter. The efficiency of a modified filter towards dimethoate removal was also quantified as the pesticide uptake.

For both sets of experiments, the efficiency of adsorption was measured by dimethoate uptake calculated as Uptake = 100% × (*C*_0_ − *C*_eq_)/*C*_0_, where *C*_0_ was the starting concentration of dimethoate. It is important to note that we confirmed that there was no decomposition of dimethoate under experimental conditions. Hence, the reduction in dimethoate concentration was solely due to the adsorption by studied ACFs.

For UPLC measurement, an ACQUITY UPLC system (Waters, Milford, MA, USA) with a tunable UV detector, controlled by the Empower software, was used. The analyses were performed using an ACQUITY UPLC™ BEH C18 column (1.7 μm, 100 mm × 2.1 mm, Waters, USA) under isocratic conditions with a mobile phase consisting of 10% acetonitrile and 90% water (*v*/*v*). The eluent flow rate was 0.2 cm^3^ min^−1^, and the injection volume was 10 mm^3^. Under these experimental conditions, the retention time of dimethoate was 2.6 min (see Figure S7, Supplementary File S1). Dimethoate was detected at 200 nm.

### 3.4. Toxicity Testing

AChE activity was assayed according to modified Ellman’s procedure [40]. Briefly, the in vitro experiments were performed by exposing 2.5 IU of commercially purified AChE from electric eel to treated OP solutions obtained in adsorption experiments (filtered supernatants in batch experiments, or filtrates in dynamic adsorption experiments) at 37 °C in 50 mM PB pH 8.0 (final volume 0.650 cm^3^). The enzymatic reaction was started by adding acetylcholine-iodide in combination with 5,5′-dithiobis(2-nitrobenzoic acid) (DTNB) as a chromogenic reagent and proceeded to proceed for 8 min. Then, it was stopped by adding 10% sodium dodecyl sulfate. Thiocholine, which was the enzymatic reaction product, reacted with DTNB and formed 5-thio-2-nitrobenzoate, whose absorbance was measured at 412 nm. AChE concentration was kept constant in all experiments and previously optimized to give an optimal spectrophotometric signal. The toxicity of treated water samples was quantified via the AChE inhibition given as AChE inhibition (%) = 100% × (*A*_0_ − *A*)/*A*, where *A*_0_ and *A* stand for the AChE activity in the absence of OP (control) and the one measured after the exposure to a dimethoate solution, respectively.

### 3.5. Semi-Empirical Quantum Chemical Calculations

Semi-empirical calculations were performed using MOPAC2016 code [41] with PM 7 method [42]. Full structural relaxation was carried out. The analysis was performed in the presence of water as a solvent. The solvent was included in the analysis implicitly, using the conductor-like screening model (COSMO) method [43]. The stoichiometry of the considered model was C_58_H_33_O_6_.

### 3.6. Adsorbent Regeneration

The adsorbent regeneration was achieved by heating at 550 °C in the He atmosphere, with a heating rate of 10 °C min^−1^. After reaching the final temperature, the adsorbent was allowed to cool down naturally. TG-DTA analysis was carried out at a heating rate of 10 °C min^−1^ up to 550 °C, under purging helium gas (Messer Serbia, 99.999%) at a flow rate of 75 mL min^−1^, using a TA Instruments Model SDT 2960 thermoanalytical device. FTIR spectra of DAHP-0.5 with dimethoate adsorbed and after regeneration was performed as explained in Section 3.2. Adsorption measurements on regenerated adsorbent were performed as explained in Section 3.3.

## 4. Conclusions

A series of ACFs was produced upon impregnating viscose fibers with different amounts of DAHP. As the concentration of DAHP increased, O and P content, SSA, and *V*_tot_ were also found to increase. As a result, the SSA of studied ACFs varied between 1000 and 2700 m^2^ g^−1^. These materials were studied as adsorbents for dimethoate; high dimethoate uptake was found for all materials, even in dimethoate solutions with a concentration as high as 5 × 10^−4^ moldm^−3^. Furthermore, the materials performed excellently in deionized water solutions and spiked tap water samples, suggesting that matrix effects were minor, while the high efficiency of dimethoate removal was also confirmed under dynamic (filtration) experiments. The latter point, connected with the fact that toxicity of water samples was significantly reduced upon the treatment, suggests that studied ACFs had a high potential for implementation into the water purification systems. This related specifically to ACFs produced with low concentrations of DAHP as impregnation agent, which performed the best as adsorbents for dimethoate and had the highest production yield. Namely, with low concentrations of DAHP as an impregnation agent, up to 20 times more ACFs were being produced than with no DAHP; DAHP had a crucial role in preventing product losses during the activation step. This point is extremely important for the rationalization and economization of the application of viscose-derived ACFs, from ACFs production to their use as adsorbents. Another important point is that adsorbents (demonstrated for the best performing material in the series) could be effectively regenerated by heating up to 550 °C and re-used without performance loss. We found that SSA was not the key factor for efficient dimethoate uptake. Instead, we found simple linear regression connecting C and O content; total pore volume could be used to reliably predict dimethoate uptake in the studied series ACFs. It is suggested that a balance between pore size distribution, carbon content, and hydrophilicity of the ACFs surface (linked to O content and directly influenced by the amount of incorporated P) led to the maximum performance in dimethoate removal from contaminated water.

## Figures and Tables

**Table 1:** Elemental composition of studied ACFs obtained using EDX(averaged over four individual spot measurements), and specific surface areas (SSA) and total pore volumes (*V*_tot_) of studied samples.

	Carbon		Oxygen		Phosphorus		Textural Properties	
DAHP-X	at.%	Δ(at.%)	at.%	Δ(at.%)	at.%	Δ(at.%)	SSA/m^2^ g^−1^	*V*_tot_/cm^3^ g^−1^
0	92.4	2.1	7.6	2.0	0	-	1932 *	0.83
0.25	91.6	3.5	8.4	3.5	0.02	0.02	1016	0.41
0.5	93.6	2.3	6.2	2.2	0.18	0.10	1250 *	0.51
1	87.9	1.3	12.0	1.3	0.11	0.04	2037	0.85
1.5	93.9	1.9	5.9	1.9	0.28	0.07	2002 *	0.85
2	91.3	2.0	7.7	2.3	0.91	0.29	2556	1.21
2.5	87.9	2.0	11.4	2.2	0.65	0.30	2018 *	0.95
5	85.6	2.5	13.1	2.7	1.32	0.34	2718 *	1.42
7.5	82.2	2.2	16.1	2.3	1.78	0.13	2763	1.83
10	77.8	5.0	19.7	5.3	1.90	0.64	2718	1.45

* ref [18].

**Table 2:** AChE inhibition before and after adsorption; Filter: adsorbent 1 mg cm^−3^, dimethoate 5 × 10^−4^ mol dm^−3^ in tap water, 1min time of filtration, 25 °C; batch: adsorbent 1 mg cm^−3^, dimethoate 5 × 10^−4^ mol dm^−3^ in tap water, 20 min contact time in batch, 25 °C.

Adsorbent	AChE Inhibition before Adsorption (% of Control)	AChE Inhibition after Adsorption in (% of Control)	
DAHP-X		Filter	Batch
0	35 ± 2	0	5 ± 1
0.25	35 ± 2	15 ± 2	5 ± 1
0.5	35 ± 2	5 ± 1	0
1	35 ± 2	5 ± 2	5 ± 1
1.5	35 ± 2	5 ± 1	5 ± 2
2	35 ± 2	5 ± 1	15 ± 2
2.5	35 ± 2	7 ± 2	12 ± 2
5	35 ± 2	9 ± 2	18 ± 2
7.5	35 ± 2	9 ± 2	20 ± 3
10	35 ± 2	12 ± 2	21 ± 2

**Table 3:** Summarized adsorption parameters for three adsorption isotherms used to fit experimental data. The sample DAHP-0.5 is emphasized as the one that shows the highest affinity towards dimethoate.

	Freundlich			Lagmuir			Dubinin–Radushkevich			
DAHP-X	*n*	*K*_f_/mg g^−1^ (mg dm^−3^)^1/n^	*R* ^2^	*q*_max_/10^3^ /mg g^−1^	*b*/dm^3^ mg^−1^	*R* ^2^	*q*_DR_/mg g^−1^	*K*_DR_/10^−7^ /mol^2^ J^−2^	*E* /kJ mol^−1^	*R* ^2^
0	1.561	29.09	0.996	3.96	0.008	0.984	186.7	1.481	1.84	0.813
0.25	1.596	18.53	0.996	2.54	0.007	0.982	134.2	2.179	1.52	0.817
0.5	1.661	35.82	0.994	4.71	0.010	0.989	196.4	1.104	2.13	0.823
1	1.652	31.88	0.995	4.12	0.009	0.987	184.2	1.239	2.01	0.822
1.5	1.411	19.11	0.998	3.11	0.006	0.972	165.1	2.490	1.42	0.799
2	1.461	17.28	0.989	5.20	0.003	0.994	149.2	2.813	1.33	0.831
2.5	1.601	21.63	0.987	4.58	0.005	0.994	151.0	2.020	1.57	0.840
5	1.485	15.99	0.988	4.90	0.003	0.995	137.9	2.959	1.30	0.836
7.5	1.369	9.91	0.997	2.33	0.004	0.997	110.2	4.389	1.07	0.804
10	1.408	9.15	0.993	2.80	0.003	0.990	99.2	4.655	1.04	0.823

**Table 4:** The results of multiple linear regression analysis where dimethoate uptake (*UPT*) under different conditions is assumed to be connected to materials properties as *UPT* (%) = *A* × at.%(C) + *B* × at.%(O) + *C* × at.%(P) + *D* × SSA + *E* × *V*_tot_.

Conditions	*A*	*B*	*C*	*D*/% g m^−2^	*E*/% g cm^−3^	*R* ^2^	Model No.
1 mg cm^−3^ ACFs, 5 × 10^−4^ moldm^−3^ dimethoate, batch	0.957	0.58	−6.1	0.006	−10.3	0.9994	1
	0.994	0.61	−7.1	/	−0.73	0.9992	2
	1.066	0.30	/	/	−8.5	0.998	3
0.1 mg cm^−3^ ACFs, 5 × 10^−6^ moldm^−3^ dimethoate, batch	0.755	0.94	−18	0.005	−4.1	0.991	4

## Data Availability

The datasets used and/or analyzed during the current study are available from the corresponding author on reasonable request.

## Supplementary Materials

The following supporting information can be downloaded online, Figure S1. EDX mapping for sample DAHP-2.5 along a single fiber; Figure S2. EDX analysis along the cross-section of the DAHP-2.5 sample; Figure S3. Representative Raman spectra of prepared ACFs with two different laser powers (2 mW, left column, and 8 mW, right column). Horizontal bars are included for easier comparison of D and G bands intensities; Figure S4. FTIR spectra (transmittance) of the investigated samples with bands assignation. Only characteristic OH vibration at 3400 cm^−1^ is seen at higher wavenumbers in all samples. The ranges where the C-P vibrations are found are also indicated; Figure S5. Dimethoate adsorption isotherms: adsorbent concentration 0.1 mg cm^−3^, 20 min contact time in batch, 25 °C; Table S1. pH values of adsorbent+dimethoate dispersion in batch experiments (adsorbent 1 mg cm^−3^, dimethoate 5 × 10^−4^ mol dm^−3^); Figure S6. Dimethoate molecule with indicated lateral dimensions; Figure S7. UPLC chromatogram of dimethoate (5 × 10^−4^ mol dm^−3^).

## Sample Availability

Samples of the carbon materials are available from the authors.
