# Supplementary material for: Viscose-Derived Activated Carbons Fibers as Highly Efficient Adsorbents for Dimethoate Removal from Water
Source: Molecules. 2022 Feb 22;27(5):1477. doi: 10.3390/molecules27051477 (PMC8911764; doi:10.3390/molecules27051477)
Supplement: Supplementary file 1 [file molecules-27-01477-s001.zip › molecules-1581753-supplementary.pdf]

Viscose-Derived Activated Carbons Fibers as Highly Efficient Adsorbents for Dimethoate Removal from Water

Ana Jocić [a], Stefan Breitenbach [b,c], DanicaBajuk-Bogdanović [d], Igor A. Pašti [d], ChristophUnterweger [b], Christian Fürst [b], TamaraLazarević-Pašti [a,*]

[a]University of Belgrade, VINČA Institute of Nuclear Sciences - National Institute of the Republic of Serbia, Mike PetrovicaAlasa 12-14, 11000 Belgrade, Serbia.

[b]Wood K plus - KompetenzzentrumHolz GmbH, Altenberger Strasse 69, 4040 Linz, Austria

[c]Institute of Chemical Technology of Inorganic Materials (TIM), Johannes Kepler University Linz, AltenbergerStrasse 69, 4040 Linz, Austria

[d]University of Belgrade – Faculty of Physical Chemistry, Studentskitrg 12-16, 11158 Belgrade, Serbia

***Corresponding author:** lazarevictlj@yahoo.com, tamara@vin.bg.ac.rs

S1. Elemental composition of produced ACFs

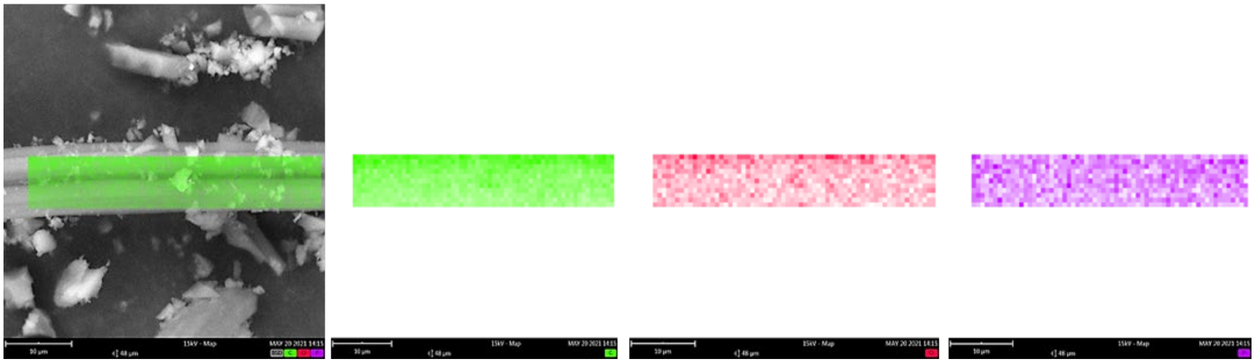

Figure S1. EDX mapping for sample DAHP-2.5 along a single fiber (scale bar 10 μm)

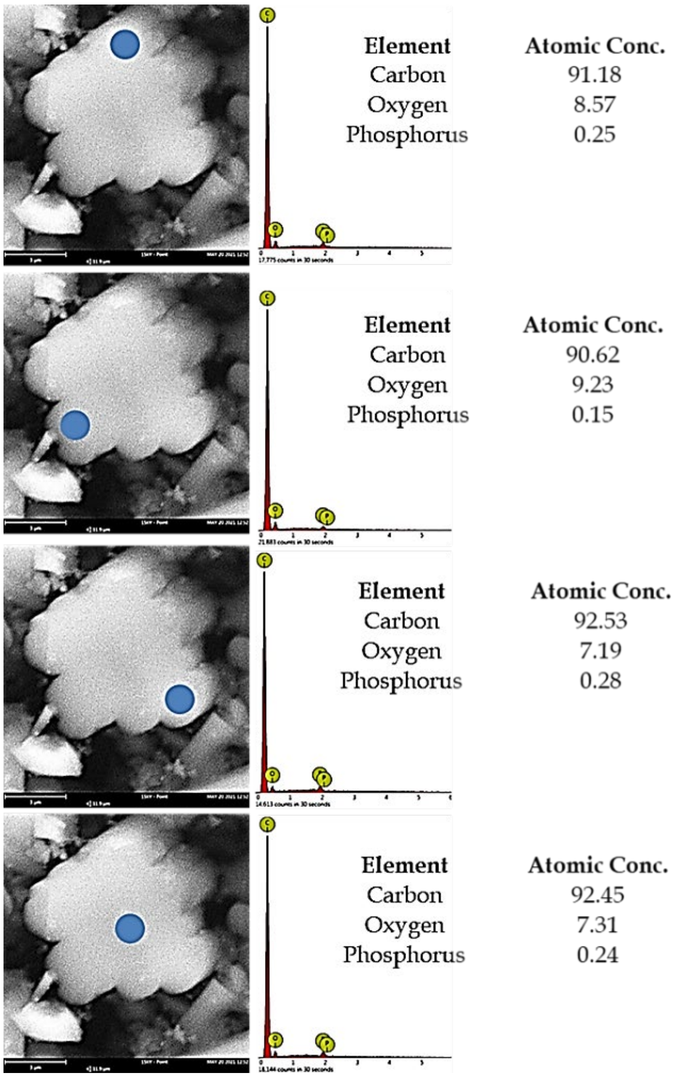

Element	Atomic Conc.
Carbon	91.18
Oxygen	8.57
Phosphorus	0.25

Element	Atomic Conc.
Carbon	90.62
Oxygen	9.23
Phosphorus	0.15

Element	Atomic Conc.
Carbon	92.53
Oxygen	7.19
Phosphorus	0.28

Element	Atomic Conc.
Carbon	92.45
Oxygen	7.31
Phosphorus	0.24

Figure S2. EDX analysis along the cross-section of the DAHP-2.5 sample (scale bar 1μm)

S2. Raman and FTIR spectroscopy

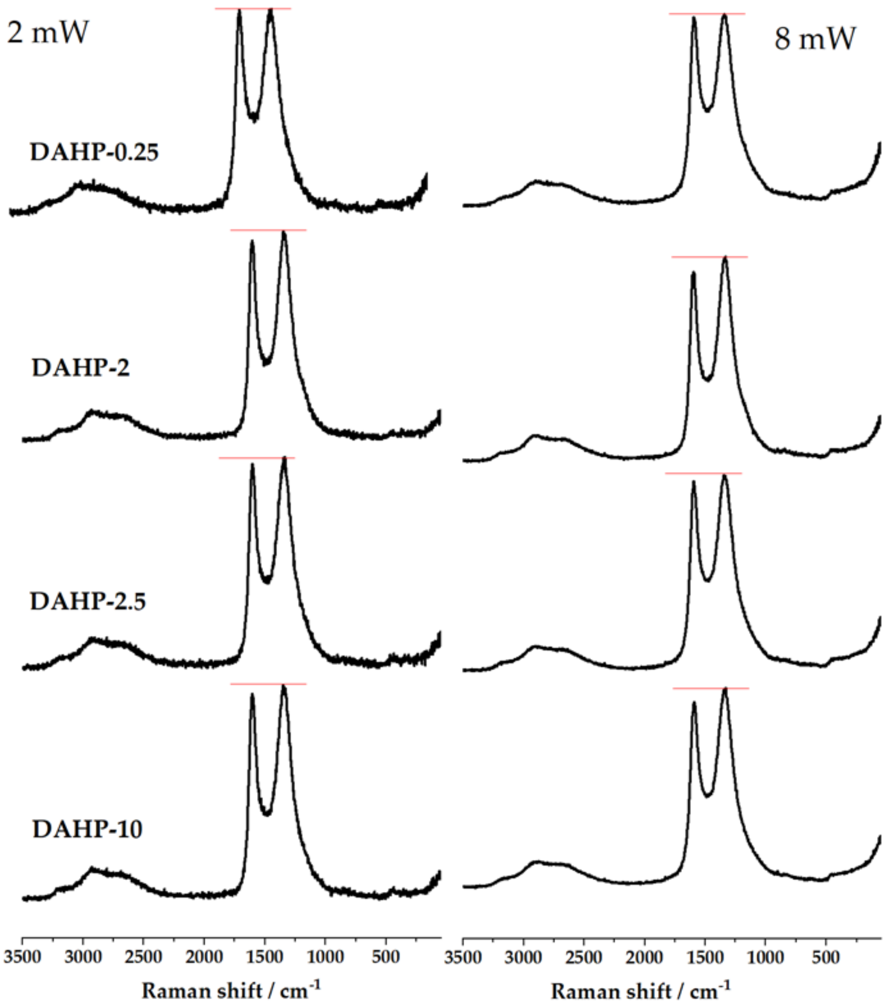

Figure S3. Representative Raman spectra of prepared ACFs with two different laser powers (2 mW, left column, and 8 mW, right column). Horizontal bars are included for easier comparison of D and G bands intensities.

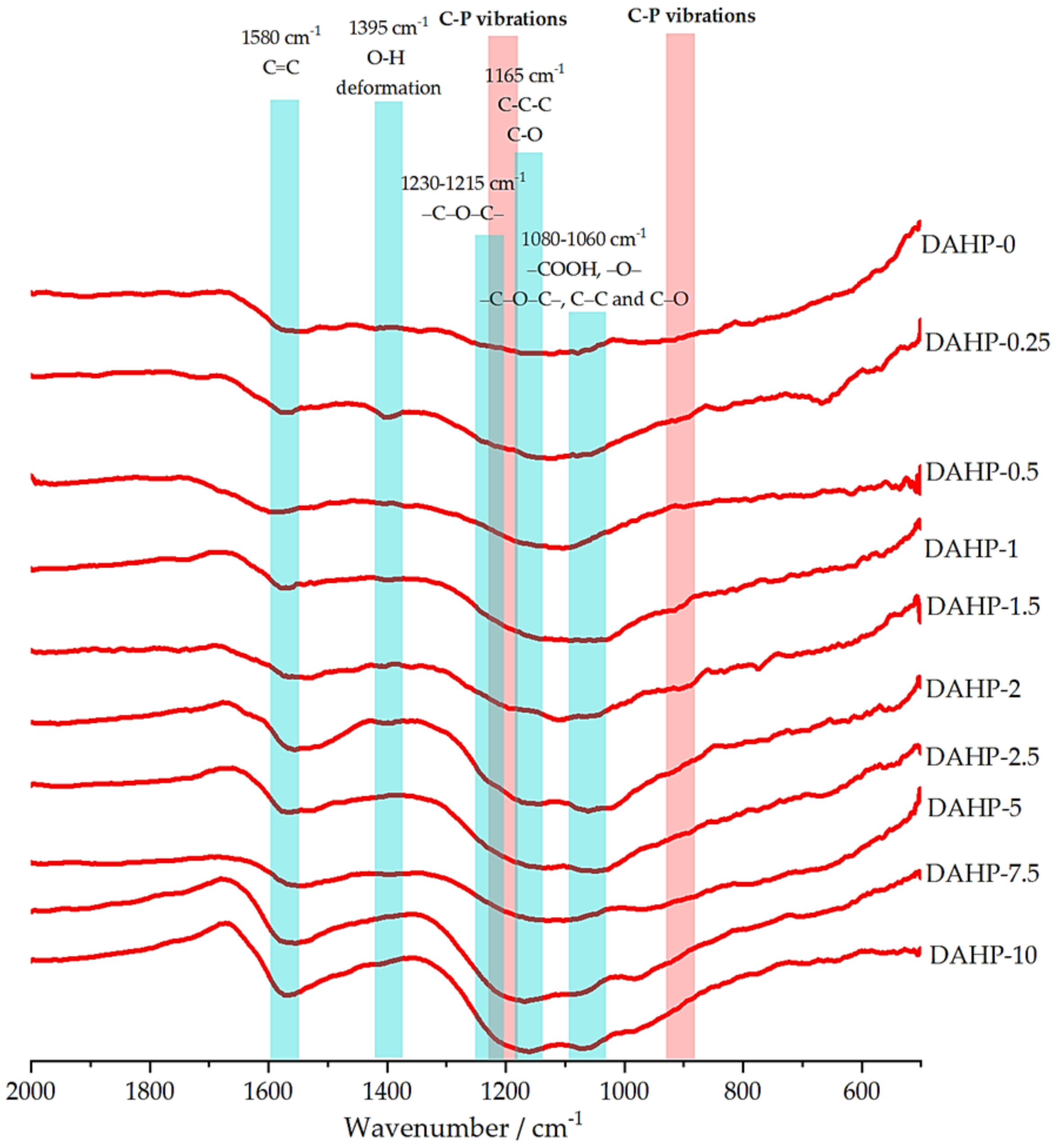

Figure S4. FTIR spectra (transmittance) of the investigated samples with bands assignation. At higher wavenumbers, only characteristic OH vibration at 3400 cm^{-1} is seen in all samples. The ranges where the C-P vibrations are found are also indicated.

S3. Dimethoate removal from aqueous solutions

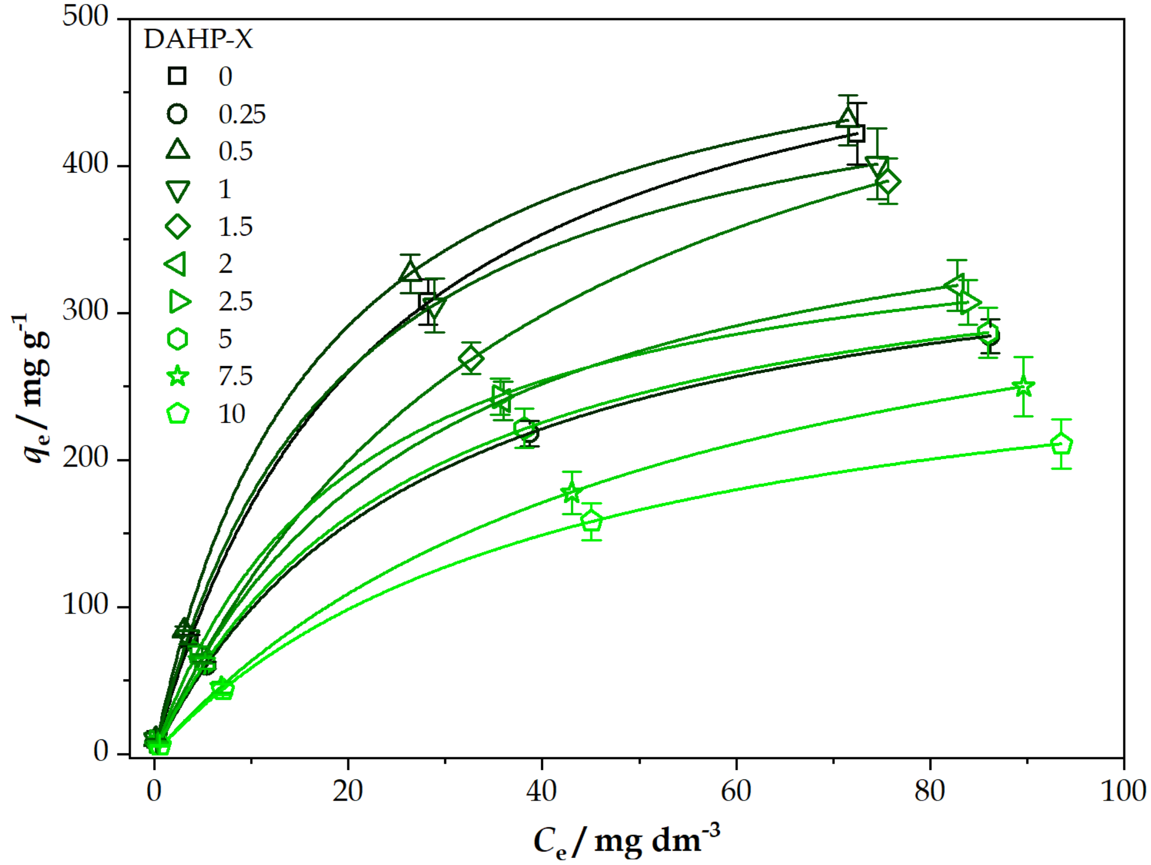

Figure S5. Dimethoate adsorption isotherms: adsorbent concentration 0.1 mg cm^{-3}, 20 min contact time in batch, 25 °C

Table S1. pH values of adsorbent+dimethoate dispersion in batch experiments (adsorbent 1 mg cm^{-3}, dimethoate 5×10^{-4} mol dm^{-3})

**DAHP-X**	**pH**
0	6.1±0.1
0.25	6.1±0.1
**0.5**	6.0±0.1
1	6.1±0.1
1.5	6.1±0.1
2	6.1±0.1
2.5	6.0±0.1
5	6.0±0.1
7.5	6.0±0.1
10	6.0±0.1

S4. Materials properties and their link to dimethoate removal

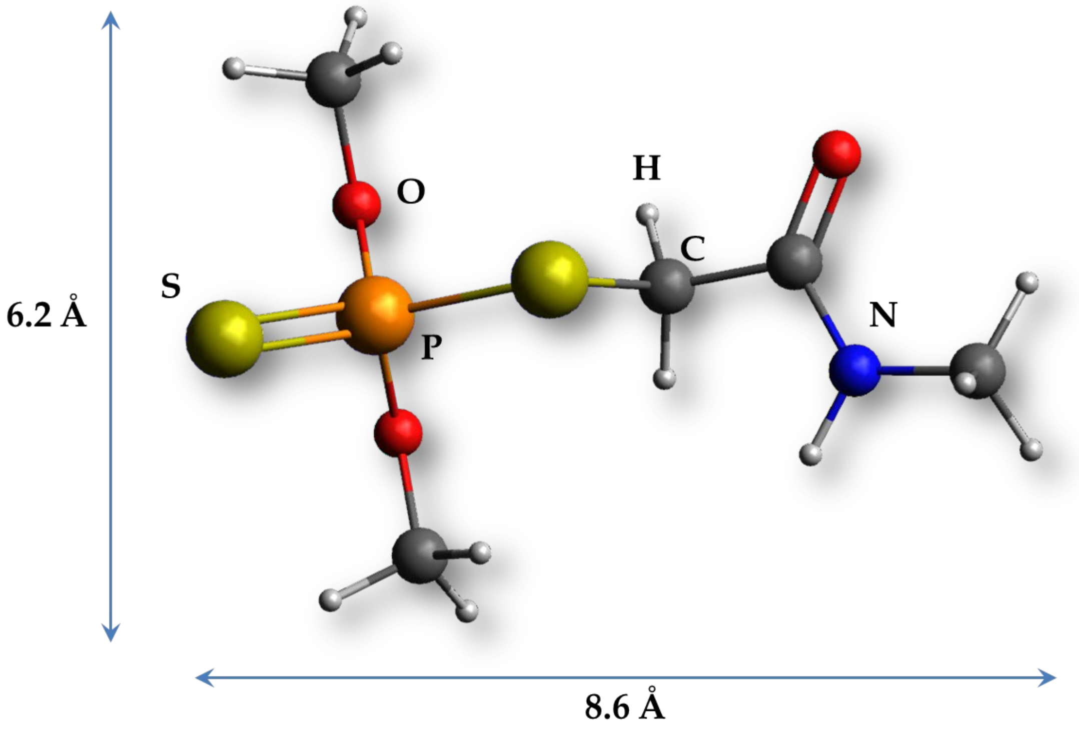

Figure S6. Dimethoate molecule with indicated lateral dimensions

S5. UPLC chromatography

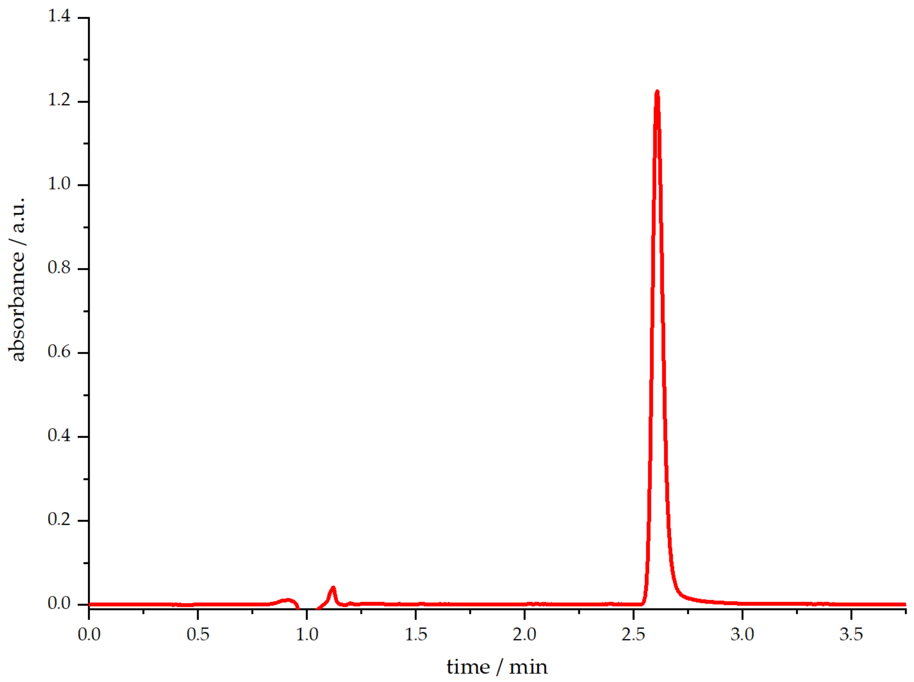

Figure S7. UPLC chromatogram of dimethoate (5×10^{-4} mol dm^{-3})
